# Maternal behaviors influence survival of ungulate neonates under heavy predation risk

**DOI:** 10.1002/ece3.70151

**Published:** 2024-08-21

**Authors:** Michael S. Muthersbaugh, Wesley W. Boone, Elizabeth A. Saldo, Alex J. Jensen, Jay Cantrell, Charles Ruth, John C. Kilgo, David S. Jachowski

**Affiliations:** ^1^ Department of Forestry and Environmental Conservation Clemson University Clemson South Carolina USA; ^2^ North Carolina Museum of Natural Sciences Raleigh North Carolina USA; ^3^ South Carolina Department of Natural Resources Columbia South Carolina USA; ^4^ USDA Forest Service Southern Research Station New Ellenton South Carolina USA

**Keywords:** deer, maternal behavior, maternal dispersion, neonate survival, predation risk, ungulate

## Abstract

Predators impose top‐down forces on prey populations, with the strength of those effects often varying over space and time and among demographic groups. In ungulates, predation risk is typically greatest for neonatal offspring, with some suggesting that predators can key in on adult activity to locate hidden neonates. However, few field studies to date have been able to directly assess the influence of maternal care on ungulate neonate survival. Using a population of white‐tailed deer under heavy coyote predation pressure, we tested the maternal dispersion hypothesis, which suggests the dispersion of maternal activity temporally and spatially attenuates risk of predation for ungulate neonates during this vulnerable altricial phase. We compared support for this hypothesis with more commonly tested hypotheses regarding the influence of habitat conditions and intrinsic factors on neonatal survival. Fawn survival to 16 weeks was 27.7%, with coyotes accounting for 59% of fawn mortalities. In support of our maternal temporal diffusion hypothesis, we found that neonatal survival decreased as more maternal visits (proportionally) occurred at night. The only other significant (*p* < .1) predictor of fawn survival was birth timing, with fawn survival decreasing as the season progressed. Given that fawn survival declined as the proportion of nighttime visits increased, and that wild pig presence and human disturbance can push doe and fawn activity toward nocturnal hours, additional research is needed to determine whether managing pig and human disturbance can decrease fawn mortality. More broadly, given the importance of recruitment in ungulate population dynamics, our finding opens a potentially important new line of inquiry on how maternal behaviors influence predation risk in large animal predator–prey ecology.

## Graphical abstract

Predators impose top‐down forces on prey populations, yet few field studies to date have been able to directly assess the influence of maternal care on ungulate offspring survival. The support we observed for the maternal dispersion hypothesis over previously widely supported hypothesized drivers of juvenile survival suggests that managers concerned with increasing neonatal survival should focus on methods to enhance the dispersive behavior of mothers during fawn rearing. More broadly, given the importance of recruitment in ungulate population dynamics, our finding of the importance of maternal behavior on juvenile survival opens a potentially important new line of inquiry for future investigations of predation risk effects in large animal predator–prey ecology.

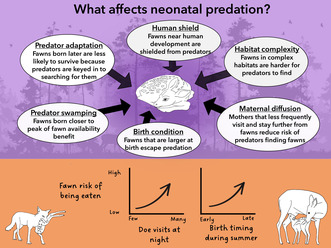

## 1 INTRODUCTION

Predators impose top‐down forces on prey populations, and understanding factors that influence the risk of predation is one of the persistent, fundamental questions in animal ecology (Abrams, 2000). Accordingly, the likelihood of being predated, or predation risk, has been extensively studied across a variety of prey species, from single‐celled ciliates (Kusch, 1993) to entire coral reef fish communities (Boaden & Kingsford, 2015). Across these studies, predation risk has generally been found to be context specific, often varying based on extrinsic conditions that vary in space (i.e., risky places) and time (i.e., risky times), leading to the encouragement of research that spans spatiotemporal scales (Moll et al., 2017; Prugh et al., 2019). Furthermore, although less frequently investigated, predation risk can vary within populations based on intrinsic individual conditions and more generally among demographic groups (Hoy et al., 2015; Pettorelli et al., 2015).

In ungulates, mortality and predation risk typically are greatest for newborn offspring (hereafter termed neonates, Caughley, 1966), and low recruitment can impact population growth (Gaillard et al., 1998). To reduce predation risk, ungulates have adopted a number of strategies. The predator swamping hypothesis states that highly synchronous reproduction by prey reduces predation risk at the individual level (Darling, 1938; Rutberg, 1987). Further, it is hypothesized that the intrinsic condition of neonates at birth impacts neonate survival, where heavier neonates may be at less risk of starvation and may need to nurse or be active less often (Therrien et al., 2008). Ungulates are also known to attempt to reduce predation risk through their birth site selection (Bowyer et al., 1999; Rearden et al., 2011). The human shield hypothesis suggests that human presence and activity alters the space use and behavior of predators, creating refugia for prey (Berger, 2007). Multiple studies have tested the structural habitat complexity hypothesis, which predicts that habitats that are structurally complex (i.e., more cover from predators) will result in lower predation risk by reducing predator foraging efficiency (Chalfoun & Martin, 2009; Crowder & Cooper, 1982). Finally, ungulates will sometimes defend young through defensive/aggressive behavior (Grovenburg et al., 2009; Smith, 1987), enabling them to survive predation attempts.

Neonate and maternal care behavior in ungulates is generally categorized into two strategies and/or phases: “hiding” or “following” (Lent, 1974). Although some ungulates are able to follow parents within hours after birth (e.g., wildebeest *Connochaetes* spp. (Estes & Estes, 1979)), most ungulates rely on crypsis to reduce the predation risk of neonates until they are large enough to effectively follow parents and flee from predators. Thus, most species have at least a short “hiding” phase for altricial neonates immediately after birth. Maternal care during the “hider” phase is characterized by infrequent nursing/socializing bouts and foraging away from their hidden offspring (Hirth, 1985). Yet, the extent to which maternal behaviors influence neonate survival remains understudied.

Researchers have described maternal behavior generally in many ungulate species, but few studies have directly measured maternal behaviors and quantified the impacts on neonate/juvenile survival. This has in large part been due to the difficulty in studying maternal behavior in a free‐ranging setting, where previous studies have been limited to direct observations in captive populations or open environments where direct observation was possible (Blank, 2017; Nuñez & Rubenstein, 2020). For example, in an experiment with captive white‐tailed deer (*Odocoileus virginianus*), Therrien et al. (2008) found that summertime food restriction impacted doe‐neonate behaviors (i.e., increased total suckling time, suckling frequency, rejected suckling attempts), and ultimately increased risk for neonate mortality. In free‐ranging mountain goats (*Oreamnos americanus*), Théoret‐Gosselin et al. (2015) observed that maternal care behaviors increased the probability of kid survival, mostly indirectly through kid mass. To our knowledge, no studies have been able to directly link the impacts of fine‐scale maternal behaviors (such as visitation rate or proximity) to predation‐related mortality in free‐ranging ungulate neonates.

Here we propose the maternal dispersion hypothesis whereby the spatial and temporal dispersion of maternal activity attenuates the risk of predation for ungulate neonates. In birds, it is generally well established that parents reduce activity at the nest in response to increased predation risk, as greater parental activity at nest sites leads to increased risk of nest predation (Fontaine & Martin, 2006; Ibáñez‐Álamo et al., 2015; Muchai & du Plessis, 2005). Similarly, predators of ungulates may use behavioral cues from mothers to help locate neonates (Chitwood, Lashley, Kilgo, Pollock, et al., 2015), and neonate activity (largely restricted to periods when mothers visit offspring) can make neonates more conspicuous and increase the chances of being detected by predators (Lingle et al., 2005; Palm, 2000). Accordingly, under the maternal dispersion hypothesis, we predicted that ungulate mothers with neonates in the “hider” phase should spend less time with their offspring, spend more time farther away from their offspring, and visit them less frequently during times of high predation risk (i.e., risky times).

To test these maternal diffusion hypotheses in a free‐ranging ungulate population, we simultaneously monitored white‐tailed deer doe and neonatal fawn (hereafter referred to as fawn) space‐use and behaviors using fine‐scale GPS collar tracking to evaluate how maternal behaviors during the “hider” phase influence neonate survival in a population under heavy predation risk. In the southeastern United States, coyotes (*Canis latrans*) account for the majority of fawn predation events and overall fawn mortality (Edge et al., 2023; Gulsby et al., 2015; Jackson & Ditchkoff, 2013; Kilgo et al., 2012), to the extent that fawn predation by coyotes limits the growth of deer populations in certain regions (Chitwood, Lashley, Kilgo, Moorman, & DePerno, 2015; Kilgo et al., 2012). As coyotes are cursorial predators typically covering large (>3 km) distances across our study area in the hours leading up to finding and consuming fawns (Jensen, 2023), we predicted that coyotes would be less likely to locate the specific area where a fawn was bedded if the doe had a large home range. Similarly, we predicted that increasing the average distance between doe‐fawn pairs would be positively related to fawn survival. In addition to maternal behaviors at risky places influencing fawn survival, under the maternal dispersion hypothesis, we predicted that increased maternal visits during temporal peaks in predation activity risk (i.e., risky times) could negatively influence fawn survival. Because coyotes are largely nocturnal in the southeastern United States. (Higdon et al., 2019; Saldo et al., 2023), we predicted that fawns visited by their mothers more often at night would be at greater risk of mortality.

In addition to hypotheses related to maternal care, we evaluated support for past hypotheses widely proposed to influence neonatal survival. Under the predator swamping hypothesis, we predicted that fawn survival would be lower if a fawn was born further from the date of the peak birth pulse that season. There is also dietary evidence that coyotes rapidly adapt to preferentially prey on fawns as they come available (Jensen et al., in press), so under our predator adaptation hypothesis, we predicted that fawn survival would be negatively related to date of birth (Kilgo et al., 2012). Under the human shield hypothesis, we predicted that reduced distance to paved roads and built cover from the edge of a doe's home range would be positively related to fawn survival due to reduced coyote activity (Jensen, 2023). While support has been mixed for the idea that understory vegetative cover positively influences fawn survival (Chitwood, Lashley, Kilgo, Pollock, et al., 2015; Edge et al., 2023; Kilgo et al., 2014; Shuman et al., 2017), under the structural habitat complexity hypothesis, we predicted that fawn survival in our system would be positively related to increasing edge density because it could slow coyote prey search efforts (Gulsby et al., 2017; Rohm et al., 2007). Finally, we predicted that birth weight would interact with visitation rate to influence risk of mortality, such that larger fawns visited less often would be at lower risk of mortality compared to smaller fawns visited more often. Collectively, an understanding of how these factors individually and interactively impact neonate survival could help guide new directions of research on predator–prey dynamics and inform potential management actions aimed at increasing ungulate neonate survival.

## 2 METHODS

### 2.1 Study area

**FIGURE 1:**
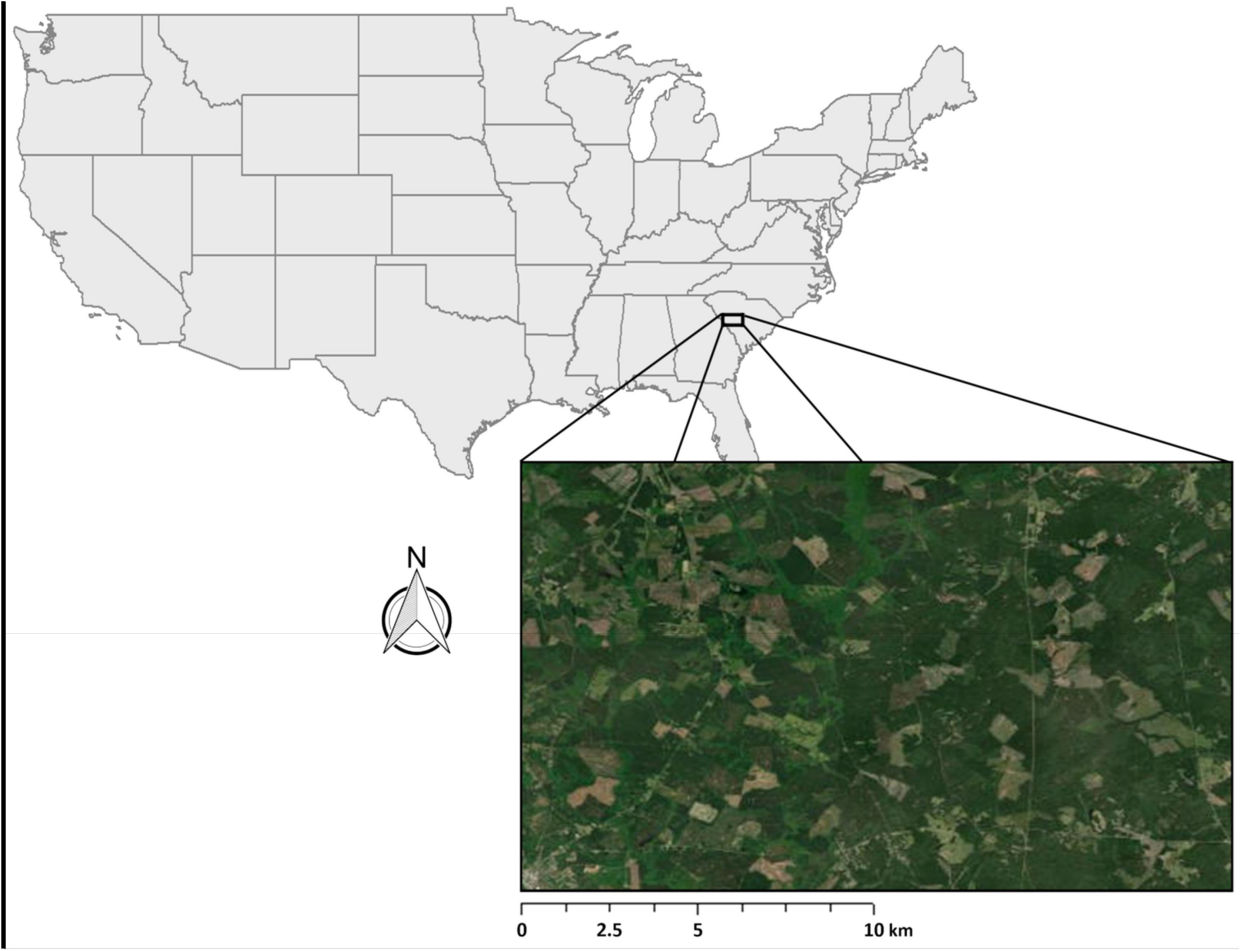
This study occurred in the Piedmont physiographic province of South Carolina. The area was characterized by rolling hills, clay‐dominated soils, planted pine stands, hardwood forests in riparian corridors, and interspersed agricultural fields. The majority of the area was managed for timber production, resulting in a mosaic of stand ages ranging from recent clearcuts to harvest‐ready forests. Human density was low (~68.8 persons/km2) throughout the study.

We studied fawn survival in a population of white‐tailed deer on ~60.7 km^2^ of privately owned lands in McCormick County, South Carolina, within the Piedmont physiographic province (Figure 1). The Piedmont region is characterized by a subtropical climate, rolling hills, clay‐dominated soils, planted pine stands, riparian hardwood associations, and scattered agricultural fields (Fields, 2007). Most of the lands in our study area were maintained as various‐aged loblolly pine (*Pinus taeda*) stands managed for wood production, cleared of other common competitor tree species such as sweetgum (*Liquidambar styraciflua*). In areas where loblolly pine was not planted, riparian areas were generally dominated by closed‐canopy, mesic hardwood associations, including white oak (*Quercus alba*), southern red oak (*Q. falcata*), hickory species (*Carya* spp.), and sweetgum. Dominant understory plant species included blackberry (*Rubus allegheniensis*), muscadine (*Vitus rotundifolia*), fennel (*Eupatorium* spp.), poison ivy (*Toxicodendron radicans*), and broomsedge (*Andropogon virginicus*). McCormick County had a low human population, with a density of ~68.8 persons/km^2^ (census.gov/quickfacts/‐mccormickcountysouthcarolina), and built cover was rare within the study area. Numerous wildlife clearings or food plots were interspersed across the property and most were planted with clover (*Trifolium* spp.), sunflowers (*Helianthus*), and/or grains like sorghum (*Sorghum* spp.). Nearly all of these wildlife clearings were smaller than 2.4 ha and many were rectangular and linear with their main purpose being to provide northern bobwhite (*Colinus virginianus*) and deer forage and hunting opportunities. Most inventoried forest stands were bordered by dirt roads or paths made for timber harvest and management activities. The study area was closed to the public except for traffic along paved primary roads. Elsewhere, human activity was primarily limited to diurnal use of secondary unpaved roads during forestry and land management activities. Coyote density within our study area was estimated to be ~25 coyotes/100km^2^ during our period of study, representing the highest density of coyotes within the state of South Carolina at the time (Youngman 2023).

### 2.2 Data collection

During 2019–2021 (January–March), we captured adult (>1.5 years old) does with rocket nets and radio transmitter remote drug delivery darts (Pneu‐Dart). We chemically immobilized captured does using intramuscular injection of the drug combination commonly known as BAM (1.1 mg/kg butorphanol tartrate; 0.37 mg/kg azaperone tartrate; 0.44 mg/kg medetomidine hydrochloride; ZooPharm, Laramie). Following immobilization, we fitted adult does with GPS collars (Model G5‐2D; 4 or 8 h mortality delay; Advanced Telemetry Systems) and implanted *Neolink*/light/temperature‐activated vaginal implant transmitters (VITs; Model M3930U, Advanced Telemetry Systems). We reversed BAM by intramuscular injection of naltrexone hydrochloride (0.51 mg/kg; ZooPharm) and atipamezole hydrochloride (4.08 mg/kg; ZooPharm). Animal capture and handling was approved by the Clemson University Institutional Animal Care and Use Committee (IACUC Protocol #: 2020–042).

Due to functionality concerns regarding *Neolink* technology during 2019, in 2020 and 2021, we monitored does with implanted VITs 1–3 times daily after the first birth event to aid in early detection of VIT expulsion (Dion et al., 2019). We captured fawns from marked does ≥3 hours after VIT expulsion and used thermal imagers (Pulsar Core RXQ30V) to aid in fawn detection. We blindfolded, weighed, recorded sex, and collared each fawn with an expandable breakaway store‐on‐board GPS collar (Model W500‐EAA, Advanced Telemetry Systems) programmed with a 6 h (2019) or 4 h (2020 and 2021) mortality switch delay. We programmed each fawn and doe collar to take a GPS fix every half hour for at least the first 6 weeks of each fawn's life. We obtained fawn body mass using a polyester mesh laundry bag and a tube scale. We wore nonscented nitrile gloves to handle fawns and released fawns quickly (<5 min) at the site of capture. Additionally, we opportunistically captured fawns born to unmarked does (not collared) whilst afield during the fawning season and processed those fawns in the same way as fawns born to marked does. We tested the precision of doe GPS collars by placing a collar on the ground within heavy cover (low‐lying creek bed with 100% canopy closure), medium cover (midslope with ~50% canopy closure), and light cover (upper slope with 0% canopy closure). Collars were deployed long enough to collect at least 65 locations, after which we averaged the distance between all GPS fixes by the collars and the geolocation taken with the handheld GPS unit which we determined to be the “true” location. We found that the mean distance error was 12.1 m (SE = 0.98) for the collar in heavy cover, 13.9 m (SE = 0.97) for medium cover, and 12.9 m (SE = 1.51) for light canopy.

To determine the causes and timing of fawn mortality, we generally followed the methods used by Kilgo et al. (2012), monitoring collared fawns at 8 h intervals through 28 days of life, then once daily through August, and 1–3 times per month through November or December or until the collar batteries failed. We ascribed causes of deaths using a combination of field evidence and DNA identification of predator species (in predation‐related mortalities). We used multiple facets of field‐based evidence including feeding behavior, caching behavior, or the presence of other species‐specific sign (e.g., tracks). Where predation was suspected but evidence was scant (e.g., only a bloody fawn collar located), we searched within a 20–30 m radius around the collar for other evidence indicative of predator species, such as tracks, scat, or hair.

When carcass remains were present and predation was suspected, we searched for killing bite wounds and subcutaneous hemorrhaging. Where subcutaneous hemorrhaging was detected, we labeled those deaths as predation. We used sterile cotton swabs to collect predator DNA samples from consumption sites on carcasses, from killing bite wounds, and bite marks or saliva on collars. We collected >1 swab per individual tissue, stored swabs at room temperature in sealed bags with desiccant, and sent them to Wildlife Genetics International (WGI; Nelson, BC, CA; Chitwood et al., 2014; Kilgo et al., 2012, 2014; Shuman et al., 2017; Edge et al., 2023) for mitochondrial DNA (mtDNA) species identification. If DNA on swabs amplified (52%, 71%, and 90% success in 2019, 2020, and 2021), we considered the detected predator species as the predator for the associated fawn mortality. If field and DNA evidence were lacking (e.g., an intact collar and no predator DNA detected), we deemed the cause of those mortalities to be unknown.

### 2.3 Characterizing maternal care

We characterized maternal care and fawn behaviors during the first 3 weeks of fawn life or until fawn death using spatial data from GPS collars of does and associated fawns. Due to the autocorrelation of relocation data, we obtained doe home range sizes by estimating kernel Brownian bridge movement models using the “getverticeshr” function from the adehabitatHR package in R (Calenge, 2006; Horne et al., 2007). We estimated the first smoothing parameter using the “liker” function (range 0 – 2), used a grid = 100, and used 20.05 m for the second smoothing parameter (i.e., collar error obtained through field trials). We determined distances between a doe‐fawn pair for shared relocation times using the “distVincentyEllipsoid” function from the geosphere R package (Hijmans et al., 2022). We defined a visit as any point where the distance between a doe‐fawn pair was <50 m and the fawn collar was in motion for more than 1 minute within the previous 30 minutes. We chose a 50 m distance because the majority of fawn activity occurs during a visit/nursing bout (Hirth, 1985) and we observed that >85% of fawn activity occurred when a doe‐fawn pair was <50 m apart. We considered all visits after sunset and before sunrise to be nighttime visits and obtained sunrise and sunset times using the suncalc R package (Benoit & Elmarhraoui, 2022).

### 2.4 Characterizing intrinsic and extrinsic factors

We calculated habitat edge density (as a proxy for habitat complexity) within the 95% home range of each doe (habitat edge length/home range area) using the 10 m resolution Dynamic World data product (Brown et al., 2022). We also calculated the minimum distance between each does' home range and (a) paved roads and (b) built cover (e.g., houses and buildings) using the function “st_distance” from the sf package (Pebesma, 2018). If any paved roads or built cover occurred within a does' home range, the distance was zero. We evaluated support for our predator swamping hypothesis by calculating the absolute number of days (non‐negative day count) a fawn was born away from the birth pulse (average birth date) for a given year. Finally, we evaluated support for our predator adaptation hypothesis by including the date of birth (ordinal date) for each fawn.

### 2.5 Survival analysis

To facilitate comparisons with other fawn survival studies (e.g., Chitwood, Lashley, Kilgo, Pollock, et al., 2015; Kilgo et al., 2012) and account for delayed entry into the sample (i.e., fawns born April–June), we estimated fawn survival from birth to 16 weeks using the Kaplan–Meier estimator for all fawns captured and monitored using a daily observation interval (Bishop et al., 2008; Kaplan & Meier, 1958; Shuman et al., 2017). We also estimated fawn survival to 16 weeks using the Kaplan–Meier estimator without data from opportunistically captured fawns because some studies have found these fawns to have higher natural survival than those found at birth, likely because they are older at the time of capture (Chitwood et al., 2017; Gilbert et al., 2014).

Within an information theoretic framework (Burnham & Anderson, 2002), we evaluated support for each of our hypothesized factors affecting fawn survival in the first 3 weeks of life using Cox‐proportional hazards models (Tables S1–S3). We focused on fawn survival in the first 3 weeks of life because most fawn mortalities, especially predation‐related mortalities, occurred before 3 weeks of age (85.9% of mortalities in our study occurred within 3 weeks of birth, similar to other fawn survival studies in the southeast; e.g., Kilgo et al., 2012, Chitwood, Lashley, Kilgo, Pollock, et al., 2015), and fawns begin transitioning away from “hider” behavior into “doe‐following” behavior around 4 weeks of age (Hirth, 1985). For this analysis, we used data from doe‐fawn pairs, excluding opportunistically captured fawns and fawns that died of starvation or disease because we were primarily interested in the potential modulating effects of maternal behaviors on predation risk. Specifically, we fit models representing competing hypotheses about factors influencing fawn survival, including predictions that fawn survival was a function of birth timing (birth model), maternal behavior and predator adaptation over time (date‐dispersion model), maternal behavior and birth condition (needy fawn model), maternal behavior in both space and time (temporal, spatial, and dispersion‐detection models), predator behavior (habitat complexity, human shield, slow search and predator adaptation models), and birth timing (predator swamping model; Tables S1–S3). We also fit null and global models.

We ranked models using Akaike's information criterion correction for small sample size (AIC*c*) using the R package MuMIn (Barton, 2018) and considered only models with empirical support (ΔAIC*c* <2 and *ω*_i_ >0.25). We evaluated relative support for 13 a priori models representing hypotheses regarding the effects of maternal behaviors, coyote encounter risk, intrinsic factors of the neonate, and landscape composition on fawn survival (Tables S1–S3). We excluded fawns that lived <1 day (*n* = 3) from Cox‐proportional analyses due to concerns about minimal data (i.e., daily averages) and because Cox‐proportional analyses are sensitive to outliers (Luo et al., 2022). Prior to analyses, we tested for collinearity among predictors and withheld highly correlated predictors (*r* ≥ |0.6|) from analysis. For the best‐supported model(s), we assessed the proportional hazards assumption using the “cox.zph” function. If a variable hazard was found to be disproportional (*p* < .05), we stratified the variable into time intervals to account for the time‐varying nature of that variable using the “survSplit” function. We interpreted variables within models to be significant if their 90% confidence intervals did not overlap zero (Sutherland et al., 2023).

## 3 RESULTS

We collared and implanted 29, 28, and 26 does with VITs, yielding 37, 26, and 20 fawns in 2019, 2020, and 2021, respectively. We captured these 83 fawns from 25 does, 18 does, and 13 does in 2019, 2020, and 2021, respectively. In addition, we opportunistically captured 10 fawns from unmarked does (2 in 2019, 6 in 2020, and 2 in 2021). Failure to capture fawns from implanted does was caused by several factors: doe collar malfunction prior to parturition (*n* = 1), death of doe prior to parturition (*n* = 3), doe was not pregnant at time of VIT implant (*n* = 1), and delayed satellite transmissions/birth alert and/or premature VIT expulsion (*n* = 22).

**FIGURE 2:**
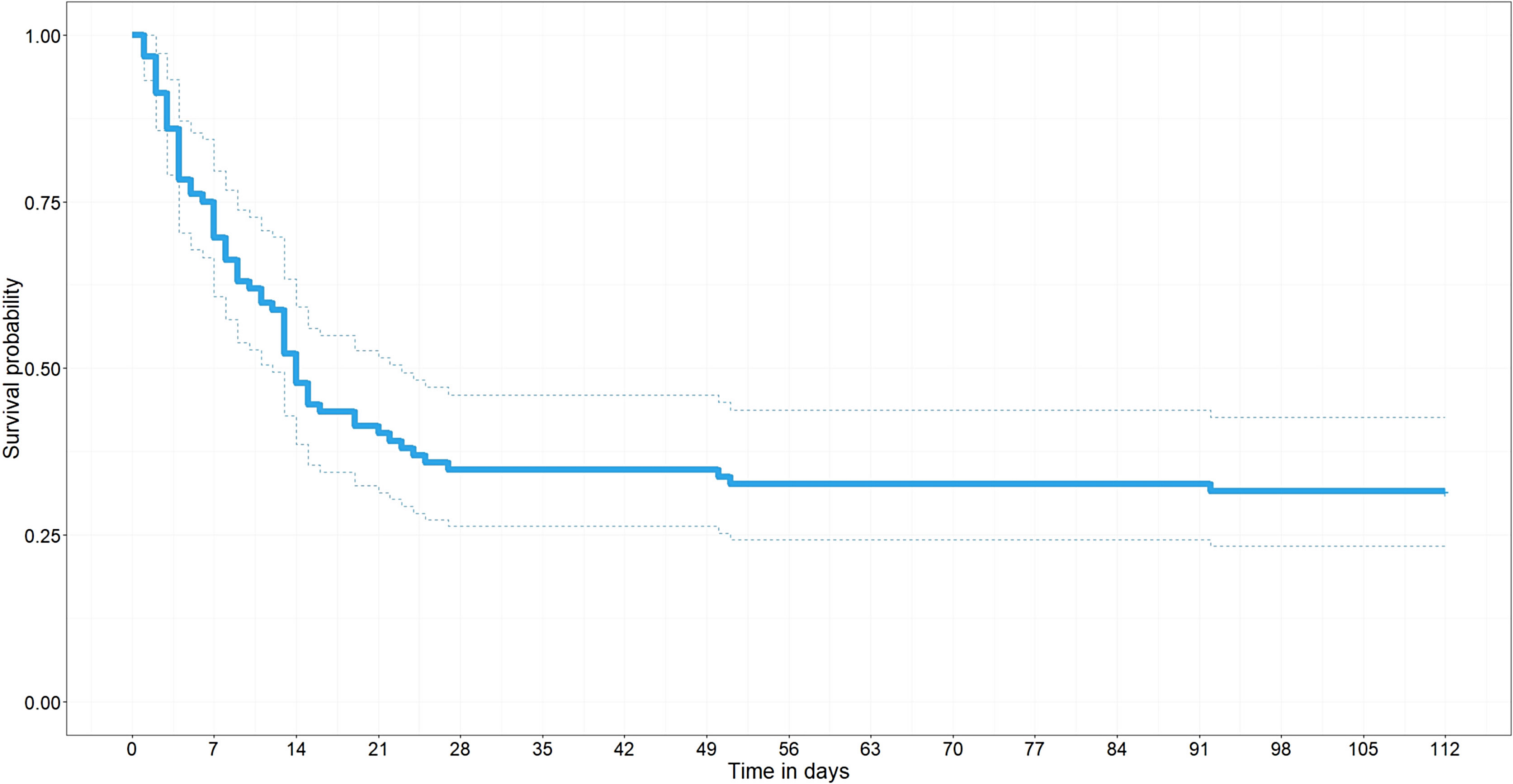
16‐week Kaplan–Meier survival curve for all fawns. Survival dropped precipitously in the first 15 days of life, after which survival began to stabilize.

For fawns from marked does, the mean date of birth across years was May 17, and was May 18, May 21, and May 9 in 2019, 2020, and 2021, respectively. Fawn mass at collaring was 2.59 ± 0.49 (SD) kg. The sex ratio for all fawns was male‐biased (56 of 93, 60.2%) and was consistent between years; 24 of 39 (61.5%) in 2019, 19 of 32 (59.4%) in 2020, and 13 of 22 (59.1%) in 2021. Average survival to 16 weeks was 0.32 (95% CI = 0.24–0.43) for all fawns (*n* = 93) and decreased through study years (0.39, 0.34, 0.18 in 2019, 2020, and 2021, respectively), but 95% confidence intervals overlapped (0.26–0.57, 0.21–0.56, 0.08–0.44 in 2019, 2020, and 2021, respectively). For fawns from only marked does (*n* = 83), survival to 16 weeks was 0.28 (95% CI = 0.20–0.39) across all study years. Fawn survival decreased precipitously in the first 15 days of life (from survival probability 1.0 to 0.4), after which survival declined more slowly (Figure 2). Of the 93 fawns, 64 died and predation was the leading cause of mortality (*n* = 47, 73.4%). Coyote predation accounted for most of the predation‐related mortality (*n* = 38, 80.9%), followed by bobcat (*Lynx rufus*) predation (*n* = 7, 14.9%), domestic dog predation (*n* = 1, 2.1%), and gray fox (*Urocyon cinereoargenteus*) predation (*n* = 1, 2.1%). Other sources of mortality included starvation (*n* = 8, 12.5%), disease/starvation (*n* = 4, 6.3%), unknown (*n* = 4, 6.3%), and vehicle collision (*n* = 1, 1.6%; Table 1).

**TABLE 1:** Number of surviving and deceased fawns and their causes of mortality from all study years for 83 fawns born to marked does and 10 opportunistically captured fawns born to unmarked does (2019–2021) in southwestern South Carolina.

Category	Cause of mortality	*n*	Percent
Surviving		29	31.2%
Predation	Bobcat	7	7.5%
	Coyote	38	40.9%
	Dog	1	1.1%
	Gray fox	1	1.1%
Natural	Injury/disease	1	1.1%
	Disease	1	1.1%
	Disease/Starvation	2	2.2%
	Starvation	8	8.6%
Other	Unknown	4	4.3%
	Vehicle	1	1.1%
	Total	93	

**FIGURE 3:**
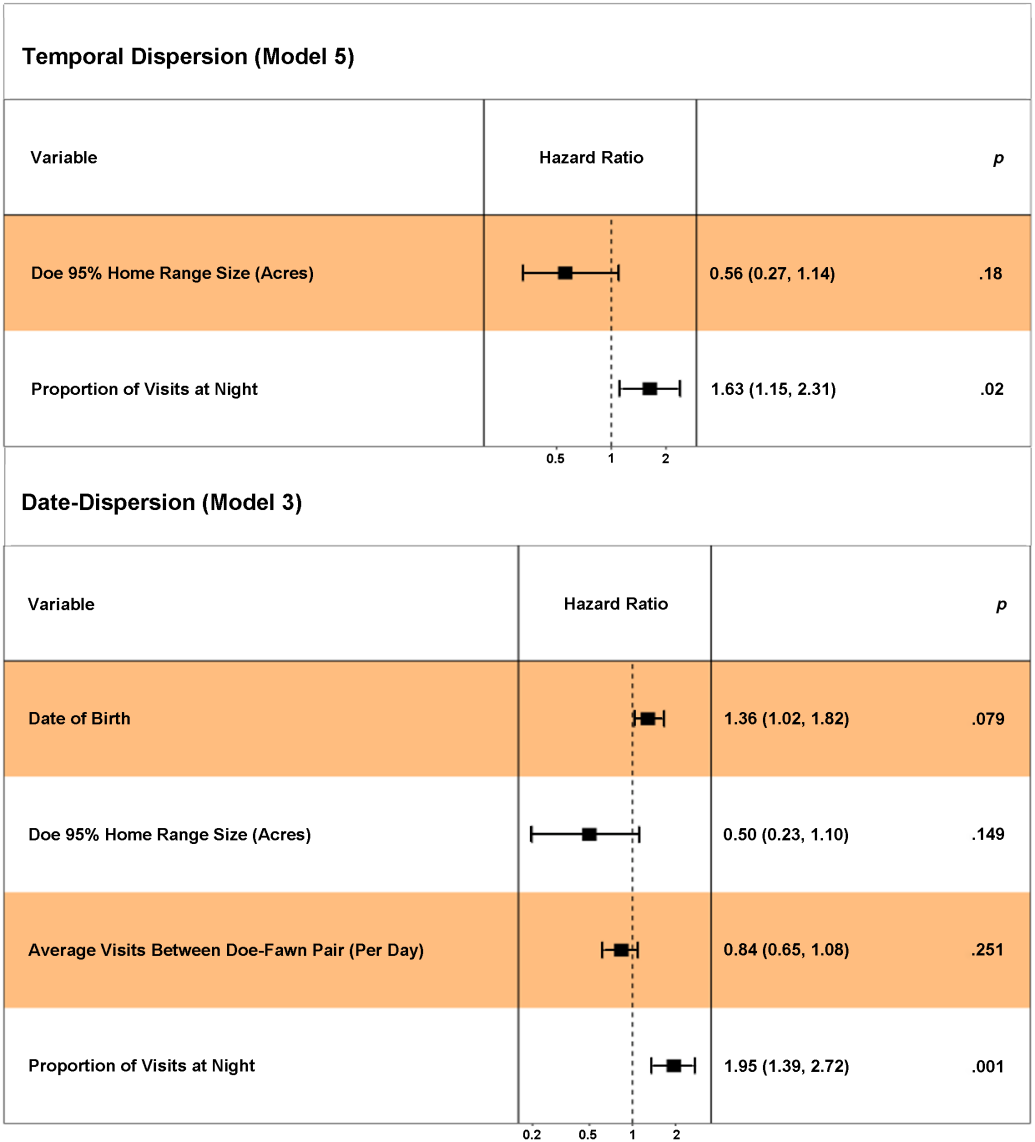
Forest plots showing the Cox‐proportional hazard estimates and 90% confidence intervals for variables in the two competing models (ΔAIC*c* <2 and *ω*_i_ >0.25), which included the temporal dispersion model (model 5, ΔAIC*c* = 0.00, *ω*_i_ = 0.52) and the date‐dispersion model (model 3, ΔAIC*c* = 1.38, *ω*_i_ = 0.26). Hazard ratio values above 1 indicate a factor is predicted to have a positive effect on the hazard (risk of mortality), therefore increases in the factor's value are associated with decreased fawn survival. Conversely, hazard ratio values below 1 indicate that an increase in the value of that factor would be associated with increased fawn survival. When 90% confidence intervals crossed 1, a positive or negative relationship with fawn survival could not be discerned. Description of models can be found in Table 1 and Tables S1–S3.

Data from 65 fawns (born to collared does and living more than 24 h) were available for the Cox‐proportional hazards analysis. Two models showed competing support (ΔAIC*c* <2 and *ω*_i_ >0.25) and indicated that maternal behaviors impacted fawn survival (Table 2). The temporal dispersion model indicated that a greater proportion of doe‐fawn visits occurring at night was associated with a higher risk of fawn mortality (Figure 3). However, we found nighttime visitation was disproportional, thus we stratified this variable and found that greater nighttime visitation was associated with a greater risk of mortality for fawns during the first 7 days of life (“time = 1”) but was not associated with risk of mortality during days 8–21 of life (“time = 2”; Tables S2 and S3). The competing date‐dispersion model similarly indicated that a greater proportion of doe‐fawn visits occurring at night was associated with a higher risk of fawn mortality (Figure 3). Date of birth was also an important predictor of fawn survival, with fawn survival decreasing as date of birth increased (Figure 3; Table S3).

**TABLE 2:** Model ranking of a priori models used to evaluate support for competing hypotheses about factors influencing fawn survival.

Model name	Model	*K*	logLik	ΔAIC*c*	*ω*_i_
(5) Temporal dispersion	Doe 95% home range size + Proportion of visits at night	2	−139.98	0.00	0.52
(3) Date‐dispersion	Date of birth + Doe 95% home range size + Average visits between doe‐fawn pair + Proportion of visits at night	4	−138.24	1.38	0.26
(7) Dispersion‐detection	Doe 95% home range size + Average visits between doe‐fawn pair	2	−142.28	4.58	0.05
(6) Spatial dispersion	Doe 95% home range size + Average distance between doe‐fawn pair	2	−142.31	4.66	0.05
(13) Null	Null	0	−144.79	5.26	0.04
(11) Slow search	Edge density in home range	1	−144.08	5.97	0.03
(9) Predator adaptation	Date of birth	1	−144.62	7.04	0.02
(12) Year	Year	1	−144.77	7.35	0.01
(10) Predator swamping	Absolute difference between date of birth and annual average birth date	1	−144.23	7.37	0.01
(8) Search area	Edge density in home range + Road length in home range + Built cover in home range	3	−143.80	10.01	0.00
(2) Birth conditions	Sex + Birth weight + Date of birth	3	−144.17	10.73	0.00
(4) Needy fawn	Average visits between doe‐fawn pair + Birth weight + Visitation*Birth weight	3	−144.38	11.16	0.00
(1) Global	Sex + Birth weight + Doe 95% home range size + Date of birth + Year + Average distance between doe‐fawn pair + Maximum distance between doe‐fawn pair + Edge density in home range + Road length in home range + Built cover in home range + Average visits between doe‐fawn pair + Proportion of visits at night + Absolute difference between date of birth and annual average birth date	12	−131.71	20.27	0.00

*Note*: We used Cox Proportional Hazards Models within an AIC framework to test and compare hypotheses. Models possessing a ΔAIC*c* <2 and *ω*_i_ >0.25 were considered to be competing models. Data pertained to the survival of 65 fawns monitored during 2019, 2020, or 2021 in southwestern South Carolina. Description of a priori models and the hypotheses tested by each model can be found in Tables S1–S3.

## 4 DISCUSSION

Our study represents one of the first attempts to quantify the impacts of fine‐scale maternal behaviors on the survival of ungulate neonates. We found support for our maternal temporal dispersion hypothesis, similar to many bird species (Fontaine & Martin, 2006; Ibáñez‐Álamo et al., 2015; Muchai & du Plessis, 2005), whereby concentrated maternal activity when predator activity is highest can increase predation risk for ungulate offspring during the “hider” phase. We also found support for our predator adaptation hypothesis, where neonates born earlier in the birthing season had a higher probability of survival. Critically, models containing maternal behaviors outcompeted models investigating alternative commonly tested hypotheses, including human shield, habitat complexity, and predator swamping, suggesting that future studies of ungulate neonate survival would likely benefit from examining maternal behaviors.

Does typically must visit their hidden neonatal fawns to provide milk more than once per day (Hirth, 1985; Lent, 1974) and most fawn activity (e.g., standing, walking, playing) is restricted to these visits. Fawns are most conspicuous during these maternal visits (Palm, 2000) and our findings support previous studies that suggest increases in fawn activity increase the risk of predation (Byers & Byers, 1983; Costelloe & Rubenstein, 2015; Lingle et al., 2005). However, rather than doe visitation overall being correlated with increasing predation risk, we found that nighttime visitation was more closely correlated with decreased fawn survival. This finding supports past observational research that suggests does may attempt to reduce the risk of coyote predation by largely restricting fawn visits to daytime hours (Higdon et al., 2019; Kautz et al., 2022). While neonates in our system were exposed to heavy predation risk, a number of factors could contribute to does being more likely to visit fawns during the night. First, shifts toward nocturnal behavior by deer have been widely observed in areas of higher human disturbance (Abernathy et al., 2023; Kilgo et al., 1998). While our study site was closed to the public, diurnal human activity was relatively high during the summer fawn rearing period due to timber harvest and other forest management operations. Second, experimental evidence from our study area suggests that invasive wild pigs (*Sus scrofa*) outcompete deer and can increase doe and fawn activity in nocturnal hours during the summer fawn‐rearing season (Saldo et al., 2024). Thus, management of human activity and wild pig populations to reduce doe nighttime visitation of neonates are important lines of future investigation, and there is a broader need for replicated studies in other systems to better understand how intrinsic and extrinsic factors influence maternal care strategies in both space and time.

In support for our predator adaptation hypothesis, neonates born later in the year experienced decreased survival compared to those born earlier in the year. While this relationship has not been widely reported, it has been described in another South Carolina deer population. Kilgo et al. (2012) postulated that predation of neonates increased throughout the birthing season because metabolic demands on coyote mothers also increased during this period. Kilgo et al. (2012) also suggested the hunting efficacy of 1‐year‐old coyotes could be increasing throughout this period since this would be their first experience hunting during birthing season. Indeed, Jensen et al. (in press) have shown that coyote diet and movement behavior in our system exhibits a lag response to increasing weekly neonate availability, suggesting the peak predation risk extends into the decline phase of fawn availability. Thus, we believe there are likely multiplicative benefits to neonates being born earlier rather than later in the birthing season in our system.

Support for our predator adaptation, but not predator swamping, hypothesis has several potentially important management implications. Prey synchronize births to swamp predators and increases the probability of an individual offspring surviving (Darling, 1938; Rutberg, 1987). Accordingly, failure to synchronize births in a system of high risk of predation could lead to decreased neonatal survival at a population level as was likely observed in this study. While the reasons for this lack of synchronization are poorly understood, the relatively long pulse of fawn availability in our study area (~90 days compared to northern regions where births are more synchronized (30–45 days; Carstensen et al., 2009; Huggler et al., 2023)) combined with coyote behavioral plasticity has been suggested as an explanation for why coyotes kill more fawns in the southeastern United States compared to other regions (Kilgo et al., 2019; Michel et al., 2020). Given coyotes are relatively recent arrivals to the southeastern United States. (<40 years; Hody & Kays, 2018), and prey can require an extended period of time to adapt to a novel predator (Sih et al., 2010), it could take a longer period of time for white‐tailed deer does to adapt birth timing. Thus, we encourage research to assess the extent to which birth pulse timing (and other maternal behaviors) vary in other regions where deer have been exposed to coyotes for longer periods of time.

A number of factors could explain why we observed greater support for maternal care influencing fawn survival compared to other factors that have been previously linked to fawn survival in other systems. Lack of support for an effect of habitat complexity or human development (i.e., human shield) on fawn survival in our study was likely due to the high density (~25 coyotes/100 km^2^; Youngman 2023) and cursorial hunting strategy of coyotes across our entire study area (Jensen, 2023). Prey exposed to predators that utilize a cursorial hunting strategy are less likely to be able to perceive predation risk cues because those predator cues are so spatially dispersed and less reliable indicators of risk on the landscape compared to ambush predators (Preisser et al., 2007; Schmitz, 2008). Thus, even if structurally complex habitats or human development are refugia from predation risk, does raising neonatal offspring might not perceive these conditions as cues of reduced predation risk from cursorial predators like coyotes. Larger fawns can flee at an earlier age and should have a higher chance of survival, but given mortality was so early in life for many fawns, any potential benefits of these intrinsic factors were likely minimized. Likewise, we did not find support for our hypothesis that spatial dispersion of a mother's activity (larger home range) would reduce predation risk for her offspring. Coyotes ranged widely across our study area immediately prior to locating and killing neonates (Jensen, 2023), likely increasing the probability of encountering even does with small home ranges. Regardless, given the potential for coyotes to use a tactic of following does back to their hidden neonate (Jensen, 2023), we encourage finer‐scale investigations into the likely complex relationship between doe, fawn, and predator behavior leading up to a predation event. Additionally, while our study did not find support for the human shield hypothesis in an area of low human density, additional research is needed to assess this relationship in areas of higher human density.

Overall, our study provides the first support for the hypothesis that dispersive maternal behaviors positively influence the survival of ungulate neonates and suggests that many studies on ungulate neonate survival may be missing a key component—the implications of maternal behaviors. Given the importance of recruitment in ungulate population dynamics (Gaillard et al., 1998), this represents an important line of inquiry into ongoing investigations in the field of large animal predator–prey ecology. Maternal behaviors toward altricial young could manifest in different ways among populations under different habitat/environmental conditions and predator communities, and predator behavior also could vary among populations (Breck et al., 2019), making it important to test how maternal behaviors vary across different levels and types of predation risk. Fortunately, new technologies (e.g., fine‐scale tracking, proximity sensors) increasingly allow for the quantification of these maternal behaviors in the wild, with the potential to provide new insights into predator–prey dynamics and inform future management actions.

## AUTHOR CONTRIBUTIONS

**Michael S. Muthersbaugh:** Data curation (lead); investigation (lead); methodology (equal); software (lead); validation (lead); visualization (lead); writing – original draft (lead). **Wesley W. Boone:** Formal analysis (equal); software (equal); validation (equal); visualization (equal); writing – review and editing (equal). **Alex J. Jensen:** Data curation (supporting); formal analysis (supporting); investigation (supporting); writing – review and editing (equal). **Elizabeth A. Saldo:** Data curation (supporting); investigation (supporting); software (supporting); writing – review and editing (supporting). **Jay Cantrell:** Conceptualization (equal); funding acquisition (equal); investigation (supporting); project administration (supporting); supervision (supporting); writing – review and editing (supporting). **Charles Ruth:** Conceptualization (lead); funding acquisition (lead); methodology (equal); project administration (equal); resources (equal); supervision (equal); writing – review and editing (supporting). **John C. Kilgo:** Conceptualization (equal); formal analysis (supporting); investigation (supporting); methodology (supporting); resources (supporting); writing – review and editing (supporting). **David S. Jachowski:** Conceptualization (lead); funding acquisition (lead); project administration (lead); supervision (lead); writing – review and editing (equal).

## CONFLICT OF INTEREST STATEMENT

The authors declare no conflicts of interest.

### OPEN RESEARCH BADGES

This article has earned an Open Data badge for making publicly available the digitally‐shareable data necessary to reproduce the reported results.

## Supporting information


Tables S1–S3.

## Data Availability

Raw data will be provided on figshare.com following manuscript acceptance for publication.
