# Supplementary material for: Maternal behaviors influence survival of ungulate neonates under heavy predation risk
Source: Ecol Evol. 2024 Aug 21;14(8):e70151. doi: 10.1002/ece3.70151 (PMC11338655; doi:10.1002/ece3.70151)
Supplement: Supplementary file 1 — Tables S1–S3. [file ECE3-14-e70151-s001.docx]

**SUPPLEMENTAL**

Table S1: Candidate models to test hypotheses and predictions related to the impacts of maternal behaviors, fetal factors, and habitat characteristics on white-tailed deer fawn survival.

| Model Designation | Model #, Name, & Associated Hypotheses | Main Prediction(s) |  | Supporting Literature |
| --- | --- | --- | --- | --- |
| Fawn Survival ~ Sex + Birth Weight (kg) + Doe 95% Home Range Size (acres) + Date of Birth + Year + Average Distance Between Doe-Fawn Pair (per day) + Maximum Distance Between Doe-Fawn Pair + Edge Density in HR + Road Length in HR + Built Cover in HR + Average Visits Between Doe-Fawn Pair (per day) + Proportion of Visits at Night + Absolute Difference Between Date of Birth and Annual Average Birth Date | 1 – Global Model:  Maternal Dispersion,  Birth Conditions,  Predator Adaptation,  Habitat Complexity,  Human Shield | Fawn survival would be positively related to birth weight, doe home range size, and distance between doe-fawn pairs, but negatively related to increasing doe fawn visitation, date of birth, and nighttime doe-fawn activity | | (Palm 2000, Lingle et al. 2005, Carstensen et al. 2009, Fuiman et al. 2010, Kilgo et al. 2012, Chitwood et al. 2015*b*) |
| Fawn Survival ~ Sex + Birth Weight (kg) + Date of Birth | 2 – Birth Model:  Birth Conditions and Predator Adaptation | Fawn survival would be positively related to birth weight but negatively related to date of birth | | (Carstensen et al. 2009, Chitwood et al. 2015*b*) |
| Fawn Survival ~ Date of Birth + Doe 95% Home Range Size (acres) + Average Visits Between Doe-Fawn Pair (per day) + Proportion of Visits at Night | 3 – Date-Dispersion Model:  Maternal Dispersion,  Predator Adaptation | Fawn survival would be positively related to visitation rates but negatively related to date of birth, and proportion of nighttime visitation | | (Palm 2000, Lingle et al. 2005, Kilgo et al. 2012) |
| Fawn Survival ~ Average Visits Between Doe-Fawn Pair (per day) + Birth Weight (kg) + Visitation*Birth Weight | 4 – Needy Fawn Model:  Maternal Dispersion,  Birth Conditions | Fawn survival would be positively related to birth weight and lower birth weights would put fawns at greater risk of mortality with greater visitation | |  |
| Fawn Survival ~ Doe 95% Home Range Size (acres) + Proportion of Visits at Night | 5 – Temporal Dispersion Model:  Maternal Dispersion | Fawn survival would be negatively related to nighttime doe-fawn visitation rates but positively related to doe home range size | | (Palm 2000, Lingle et al. 2005, Kilgo et al. 2012) |
| Fawn Survival ~ Doe 95% Home Range Size (acres) + Average Distance Between Doe-Fawn Pair (per day) | 6 – Spatial Dispersion Model:  Maternal Dispersion | Closer doe-fawn proximity would negatively impact fawn survival | | (Palm 2000, Lingle et al. 2005) |
| Fawn Survival ~ Doe 95% Home Range Size (acres) + Average Visits Between Doe-Fawn Pair (per day) | 7 – Dispersion-Detection Model:  Maternal Dispersion | Fawn survival would be positively related to doe home range size due to a dilution effect, and increased doe-fawn visitation rates would negatively impact fawn survival due to increased detectability of active fawns | | (Palm 2000, Lingle et al. 2005, Chitwood et al. 2015*b*) |
| Fawn Survival ~ Edge Density in Home Range + Road Length in Home Range + Built Cover in Home Range | 8 – Search Area Model:  Habitat Complexity,  Human Shield | Fawn survival would be positively related to edge density due to reduced coyote foraging efficiency in more complex habitats, and positively related to paved roads and built cover due to a coyote avoidance of these areas | | (Rohm et al. 2007, Jensen 2023) |
| Fawn Survival ~ Date of Birth | 9 – Predator Adaptation Model:  Risky Late Birth | Fawns born later in the summer would have the greatest mortality risk | | (Rohm et al. 2007, Kilgo et al. 2012) |
| Fawn Survival ~ Absolute Difference Between Date of Birth and Annual Average Birth Date | 10 – Predator Swamping Model:  During peak birth, too many fawns are born for predators to kill them all | Fawns born further temporally from the annual peak birth date (average) would experience greater mortality risk | | (Darling 1938, Rutberg 1987, Michel et al. 2020) |
| Fawn Survival ~ Edge Density in Home Range | 11 – Slow Search Model:  Habitat Complexity | Edge density would positively impact fawn survival because of reduced coyote foraging efficiency in more complex habitats | | (Rohm et al. 2007) |
| Fawn Survival ~ Year | 12 – Year Model: | Fawn survival would statistically vary among study years | |  |
| Fawn Survival ~ 1 | 13 – Null Model: Fawn survival would not be related to studied factors |  | |  |

Table S2: Model output for the best supported Cox Proportional Hazards model investigating white-tailed deer (*Odocoileus virginianus*) fawn survival in southwestern South Carolina. This model sought to determine whether doe home range size (acres) or the proportion of visits between the doe and fawn that occurred at night were associated with fawn survival. This model possessed 0.53 model weight and possessed an AIC*c* 1.38 units lower than the next best, and only other competing, model. Data pertained to 65 fawns monitored during 2019, 2020, or 2021. Statistical significance (p < 0.1) is indicated by an *.

|  | coef | exp(coef) | SE(coef) | robust SE | Z | p-value |
| --- | --- | --- | --- | --- | --- | --- |
| Doe 95% Home Range Size (acres) | -0.58 | 0.56 | 0.26 | 0.44 | -1.34 | 0.18 |
| Proportion of Visits at Night time = 1 | 0.78 | 2.19 | 0.24 | 0.20 | 3.87 | 0.0001* |
| Proportion of Visits at Night time = 2 | -0.27 | 0.77 | 0.43 | 0.56 | -0.47 | 0.64 |

Table S3: Model output for the second best supported Cox Proportional Hazards model investigating white-tailed deer (*Odocoileus virginianus*) fawn survival in southwestern South Carolina. This model sought to determine whether doe home range size (acres), the proportion of visits between the doe and fawn that occurred at night, average daily visitation rates between the doe and fawn, and the date of birth were associated with fawn survival. This model possessed 0.27 model weight, a ΔAIC*c* of 1.38, and was the only other competing model. Data pertained to 65 fawns monitored during 2019, 2020, or 2021. Statistical significance (p < 0.1) is indicated by an *.

|  | coef | exp(coef) | SE(coef) | robust SE | Z | p-value |
| --- | --- | --- | --- | --- | --- | --- |
| Doe 95% Home Range Size (acres) | -0.69 | 0.50 | 0.28 | 0.48 | -1.44 | 0.15 |
| Proportion of Visits at Night time = 1 | 0.90 | 2.46 | 0.25 | 0.21 | 4.29 | 0.00002* |
| Proportion of Visits at Night time = 2 | -0.03 | 0.97 | 0.45 | 0.57 | -0.06 | 0.95 |
| Average Visits Between Doe-Fawn Pair | -0.18 | 0.84 | 0.17 | 0.16 | -1.15 | 0.25 |
| Date of Birth | 0.31 | 1.36 | 0.17 | 0.18 | 1.75 | 0.08* |
